# Large-scale analysis reveals the specific clinical and immune features of CD155 in glioma

**DOI:** 10.18632/aging.102131

**Published:** 2019-08-04

**Authors:** Fangkun Liu, Jing Huang, Yuanyuan Xiong, Shuwang Li, Zhixiong Liu

**Affiliations:** 1 Department of Neurosurgery, Xiangya Hospital, Central South University (CSU), Changsha 410008, China; 2 National Clinical Research Center for Geriatric Disorders，Xiangya Hospital, Central South University, Changsha, Hunan 410008, China; 3 Department of Psychiatry, the Second Xiangya Hospital, Central South University, Changsha, Hunan 410011, China; 4 Mental Health Institute of the Second Xiangya Hospital, Central South University, Chinese National Clinical Research Center on Mental Disorders (Xiangya), Chinese National Technology Institute on Mental Disorders, Hunan Key Laboratory of Psychiatry and Mental Health, Changsha, Hunan 410011, China

**Keywords:** CD155, checkpoint, immune response, glioma, prognosis

## Abstract

Recent studies demonstrated that CD155 plays an important role in anti-tumor immune responses. However, its role in glioma remains unclear. Here, we identify CD155 as a promising immune target in glioma. CD155 expression was significantly highly expressed in glioblastoma but not in normal brain tissue. Subsequent analysis based on genetic and clinical data from 1173 glioma patients in Rembrandt and TCGA dataset suggested that CD155 related genes of immune response were mainly positively correlated with CD155 expression. CD155 expression was positively correlated with immune-related metagenes STAT1, HCK, LCK, and MHC I but negatively associated with IgG. CD155 expression was positively correlated with biomarker gene expression of infiltrating immune cells, suggested that high CD155 expression in gliomas tend to have more infiltrating immune cells compared with gliomas with low CD155 expression. Pearson correlation analysis showed that CD155 is associated with CD96, CD226, Nectin4, PD-L1, B7-H2, NR2F6 and GITR, implying the potential synergistic effects of these checkpoint proteins. These findings implied that CD155 is a promising immunotherapy target, combined with existing immune checkpoint blockade therapies for glioma.

## INTRODUCTION

Glioma, especially high-grade glioma (HGG) is a severe malignant brain tumor associated with an extremely aggressive clinical course and poor overall survival (OS). Despite advances in neurosurgical resection, chemotherapy, and radiation, conventional intervention remains inadequate to prevent tumor recurrence and patient deterioration [1-4]. The discovery of the intracranial lymphatic system indicates that the central nervous system actively communicates with the immune system [5]. Recent years have been associated with striking success in tumor immunotherapy, especially checkpoint inhibitors that target programmed cell death protein 1 (PD-1) / programmed death ligand 1 (PD-L1), and cytotoxic T-lymphocyte-associated antigen-4 (CTLA-4) [6]. Whereas several studies on checkpoint inhibitors have been exploited and applied to clinical settings for gliomas, many patients do not respond to these therapies [6, 7]. This has catalyzed enormous interest in the identification of additional targets to increase response rates and improve therapeutic efficacy.

CD155, also referred to as necl-5, has recently been recognized as a promising target in tumor immunotherapy [8–11]. It was originally identified as a poliovirus receptor (PVR), which is broadly overexpressed in several human malignancies but weakly expressed in normal tissues [12–14]. CD155 overexpression promotes tumor cell proliferation, invasion and migration, and is correlated with enhanced tumor progression and poorer prognosis [12, 16]. Therefore, CD155 is currently been investigated in recombinant oncolytic virotherapy in different clinical trials. CD155 also belongs to nectin-like molecule family, as an immunoglobulin-like adhesion molecule, CD155 participates in cell motility, natural killer and T cell-mediated immunity. It is the ligand for immunoglobulin (Ig) superfamily members co-inhibitory receptors T-cell immunoglobulin and ITIM domain (TIGIT) and CD96 together with the co-stimulatory receptor CD226 [16, 17]. Increasing evidence found CD155 overexpression in tumor cells can induce immune escape, contributing to tumor immunosuppression. Blockade of CD155 function enhanced antitumor responses to multiple immune checkpoint blockades including PD-1, CTLA-4, TIGIT, and CD96, suggesting the clinical potential of cotargeting CD155 and these immune checkpoints [8]. Therefore, strategies targeting the interactions between CD155 and inhibitory immune cell receptors is promising in the treatment of tumors with CD155 overexpression.

As a promising target for immunotherapy, the relationship between CD155 expression and immune-related markers, cells and receptors in glioma is largely unknown. Previous studies found CD155 is up-regulated in HGG cases and in primary cell lines derived from these tumors [12, 14]. And it has been investigated as a poliovirus receptor for therapeutic intervention with oncolytic poliovirus recombinants in recurrent glioblastoma (GBM) [1]. However, comprehensive reports of CD155 expression in all gliomas are lacking so far. Therefore, we analyzed the protein level of CD155 in human glioma samples, the clinical and molecular data of 474 glioma samples from REpository for Molecular BRAin Neoplasia DaTa (Rembrandt) dataset to investigate CD155 expression, as well as its relationship with immune related molecules in glioma. To overcome the limitations of individual studies, we validated our findings using 699 glioma samples from The Cancer Genome Atlas (TCGA) dataset. Collectively, these findings would give us a better understanding of CD155 in glioma, and provide molecular basis for the development of CD155-targeted cancer immunotherapies.

## RESULTS

### CD155 expression is significantly up-regulated in GBM

**Figure 1:**
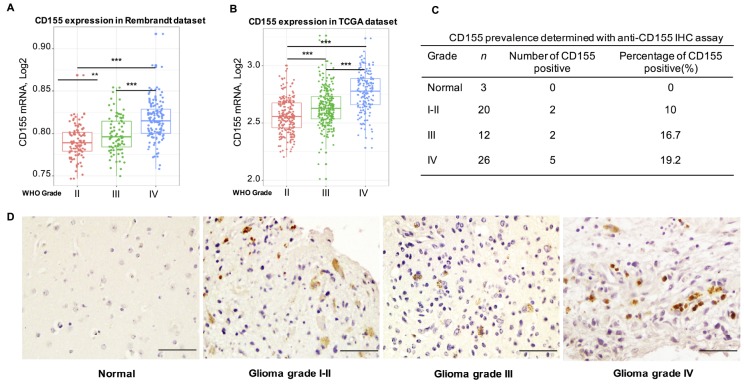
**Relationship between CD155 expression and clinical glioma parameters.** CD155 transcript levels increase with WHO grade in the Rembrandt dataset (**A**) and TCGA dataset (**B**). Protein expression pattern of CD155 in glioma showed CD155 was highly expressed in GBM at protein level (**C**–**D**). Glioma tissues of different grades were immunostained for CD155 using anti-CD155 antibody and 3,30-diaminobenzidine (DAB; brown). Haematoxylin was used for nuclear counterstaining (blue). The summarized table included number and percentage of CD155-positive samples out of number of total samples. Scale bars, 100 mm. * indicates p value < 0.05, ** indicates p value < 0.01, *** indicates p value < 0.001.

To analyze the expression of CD155 in gliomas, we extracted clinical and genetic data from Rembrandt and TCGA datasets. First, we investigated CD155 expression in gliomas based on WHO glioma grades. *CD155* expression was significantly up-regulated in higher grade gliomas; specifically, WHO grade IV glioma (GBM) showed the highest expression, as compared to that in WHO grade II and grade III gliomas from the Rembrandt dataset (Figure 1A). These results indicated that CD155 was significantly enriched in GBM. We next conducted receiver operating characteristic (ROC) curve for CD155 and GBM. The area under the curve (AUC) was 76.8% for Rembrandt dataset (Supplementary Figure 1A). Highly consistent results were obtained using the TCGA dataset (Figure 1B). The AUC of ROC curve in TCGA dataset was 77.0% (Supplementary Figure 1B). To validate CD155 upregulation in gliomas, we performed immunohistochemical analyses using human glioma samples. CD155 was not detectable in normal brain tissues, but expressed in gliomas of different grades (Figure 1C, 1D).

The transcript levels of *CD155* in different glioma subtypes were further analyzed. Being classified by differences in gene expression, subtypes of glioma are associated with different prognoses and responses to treatment [18]. Results demonstrated that *CD155* expression was much higher in mesenchymal-molecular subtype and classical subtype gliomas compared to that in the other two subtypes based on the Rembrandt dataset (Supplementary Figure 1C). Similar results were found for the TCGA dataset (Supplementary Figure 1D).

**Figure 2:**
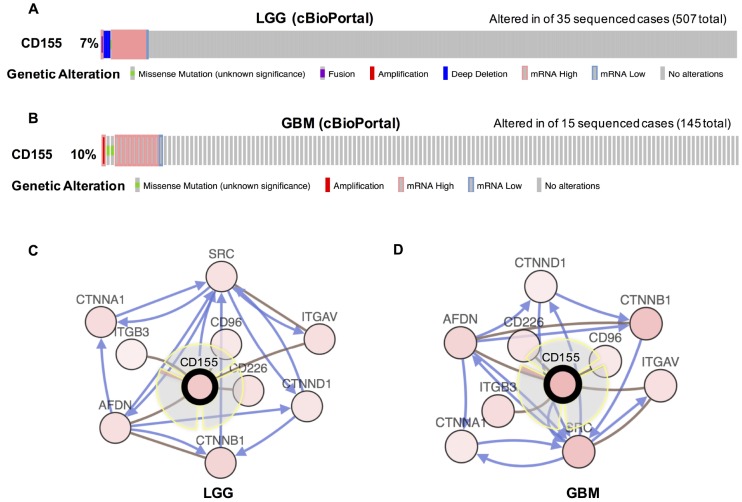
**Visual summary of CD155 alterations and biological interaction network in gliomas (cBioPortal).** An overview of genomic alterations in CD155 affecting individual samples (columns) in LGG (**A**) and GBM (**B**) from TCGA dataset was shown. Columns of different colors represent different types of genetic alterations. Results are derived from copy-number analysis algorithms like GISTIC or RAE, and indicate the copy-number level per gene. Deep Deletion indicates a deep loss, possibly a homozygous deletion. Amplification indicate a high-level amplification (more copies, often focal). Network view of the CD155 neighbor genes in LGG (**C**) and GBM (**D**) from TCGA dataset were generated. CD155 was set as seed genes (gene with black think border), and all other genes were automatically identified as altered in LGG and GBM. Derived from BioPAX: the red connection indicates that two proteins connected are members of the same complex, the blue connection indicates that the first protein controls a reaction that changes the state of the second protein.

We then evaluated the types and frequency of *CD155* alterations in gliomas based on sequencing data from glioma patients in the TCGA dataset. CD155 was altered in 35 of 570 (7%) low grade glioma patients (Figure 2A). These alterations were mRNA upregulation in 26 cases, deep deletion in 6 cases, mRNA downregulation in 2 cases, and mutation in 1 case. CD155 was altered in 14 of 145 (10%) GBM patients (Figure 2B). These alterations were mRNA upregulation in 10 cases, mutation in 2 cases, amplification in 1 case, and mRNA downregulation in 1 case. Thus, mRNA upregulation is the most common type of CD155 gene alterations in glioma. We also used cBioPortal (https://www.cbioportal.org) to see the biological interaction network of CD155 in gliomas. CD155 can form the same complex with CD96, CD226, AFDN, ITGAV, ITGB3, and AFDN in low-grade glioma (LGG) (Figure 2C) and GBM samples (Figure 2D) derived from the Biological Pathway Ex-change (BioPAX).

### CD155 is closely related to immune functions in glioma

**Figure 3:**
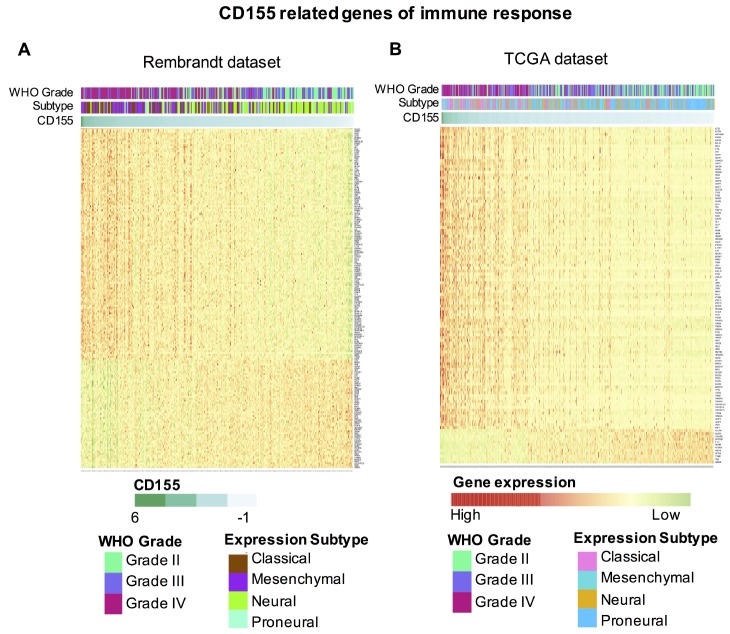
**Heatmap analysis of the relationship between CD155 and immune function-related genes in glioma.** The relationship between CD155 and glioma grade, molecular subtypes, and immune function-related genes are presented. Data were downloaded from the AmiGO 2 website for Rembrandt (**A**) and TCGA (**B**) datasets.

To further explore the role of CD155 in immune functions in gliomas, we downloaded gene sets related to immune system from the AmiGO 2 website (http://amigo.geneontology.org/amigo). Genes significantly related to *CD155* (|R| > 0.4 and p < 0.05) based on Rembrandt and TCGA datasets were selected for heatmap analysis. In total, 178 immune-related genes in Rembrandt and 116 immune-related genes in TCGA datasets were defined. 121 genes included were significantly positively correlated with *CD155* expression in Rembrandt dataset, 104 genes included were significantly positively correlated with *CD155* expression in TCGA dataset (Figure 3A, 3B). A detailed list of these genes based on the two datasets is presented in Supplementary Table 1. Thus, the expression pattern of *CD155* was found to be strongly linked with immune responses in glioma.

### CD155-related inflammatory activities

**Figure 4:**
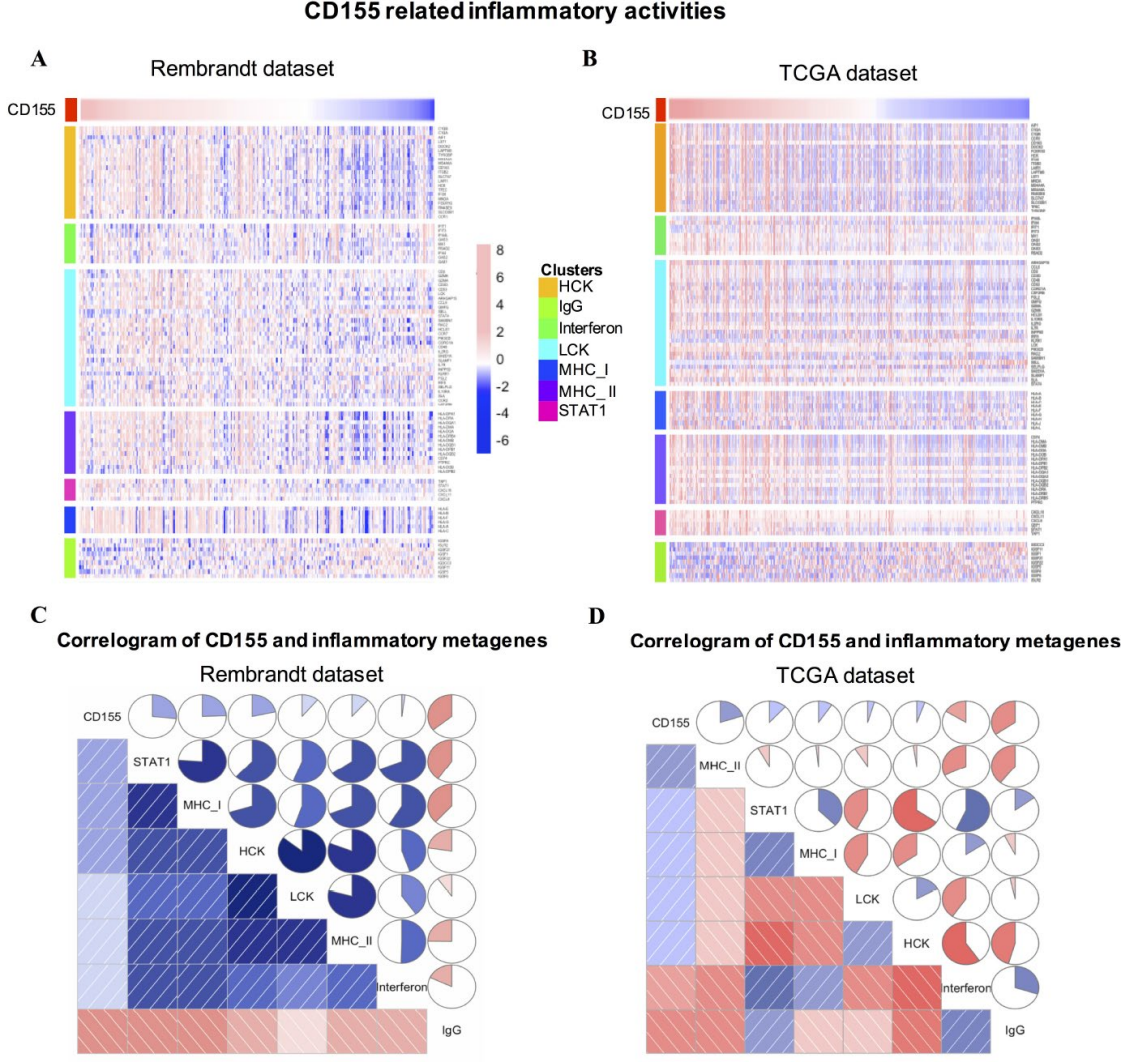
**CD155-related inflammatory activities in gliomas.** The relationship between CD155 and seven metagenes in are Rembrandt (**A**) and TCGA (**B**) datasets were presented as a heatmap. Correlogram showed the correlation between CD155 and seven immune-related metagenes based on Rembrandt (**C**) and TCGA (**D**) datasets.

In order to understand *CD155*-related inflammatory activities, we chose seven metagenes including 104 genes related to different types of inflammation and immune responses [19]. A detailed list of these genes is summarized in Supplementary Table 2. Based on Rembrandt and TCGA datasets, most clusters were positively associated with *CD155* expression (Figure 4A, 4B). To validate clustering results, Gene set variation analysis (GSVA) was used to transform the expression data of these metagenes into enrichment scores. Then, we generated correlograms using R language according to the Pearson correlation values based on comparisons between *CD155* and the seven metagenes (Figure 4C, 4D). GSVA results were consistent with heatmap analysis. *CD155* expression was positively correlated with STAT1, MHC I, and MHC II in the Rembrandt and TCGA datasets but negatively associated with IgG, a marker for B cells.

### Relationship between CD155 and infiltrating immune cells

**Figure 5:**
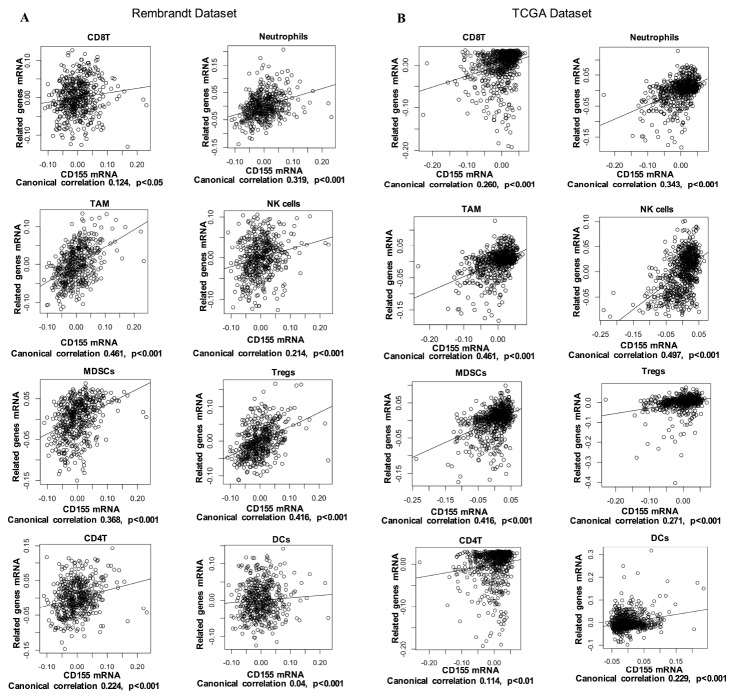
Correlation of CD155 expression with immune cell-specific marker genes in Rembrandt (**A**) and TCGA (**B**) datasets. Each open circle represents a single patient with glioma. A regression line was fitted to the dot plot.

Tumor-infiltrating immune cells play a key role in tumor development and control. In glioma, these immune cells can be important effector cells in inflammation and immune responses. Thus, we examined the correlation of *CD155* expression with immune cell-specific marker genes to evaluate the relationship between CD155 expression and immune cells. Six immune cells frequently infiltrating tumors were selected, including CD8+ T cells, CD4+ T cells, neutrophils, tumor-associated macrophages (TAMs), natural killer (NK) cells, myeloid-derived suppressor cells (MDSCs), Dendritic cells (DCs), and regulatory T cells (Tregs). The detailed information of immune cell-specific marker genes has been listed in Supplementary Table 3. As shown by Canonical correlation analyses, glioma-derived *CD155* expression was positively correlated with biomarker gene expression of all six immune cell types in both the Rembrandt and TCGA datasets (Figure 5A, 5B). We also performed immunohistochemical staining of immune cell-specific markers including CD11b, CD14, CD56, CD11c, FOXP3, CD8 and CD4 in glioma tissues. Higher expression levels of these markers can be detected in CD155-positive glioma samples compared with CD155-negative ones (Supplementary Figure 2). These results suggested that high CD155 expression in gliomas tend to have more infiltrating immune cells compared with gliomas with low CD155 expression.

### Correlation between CD155 and immune checkpoints

**Figure 6:**
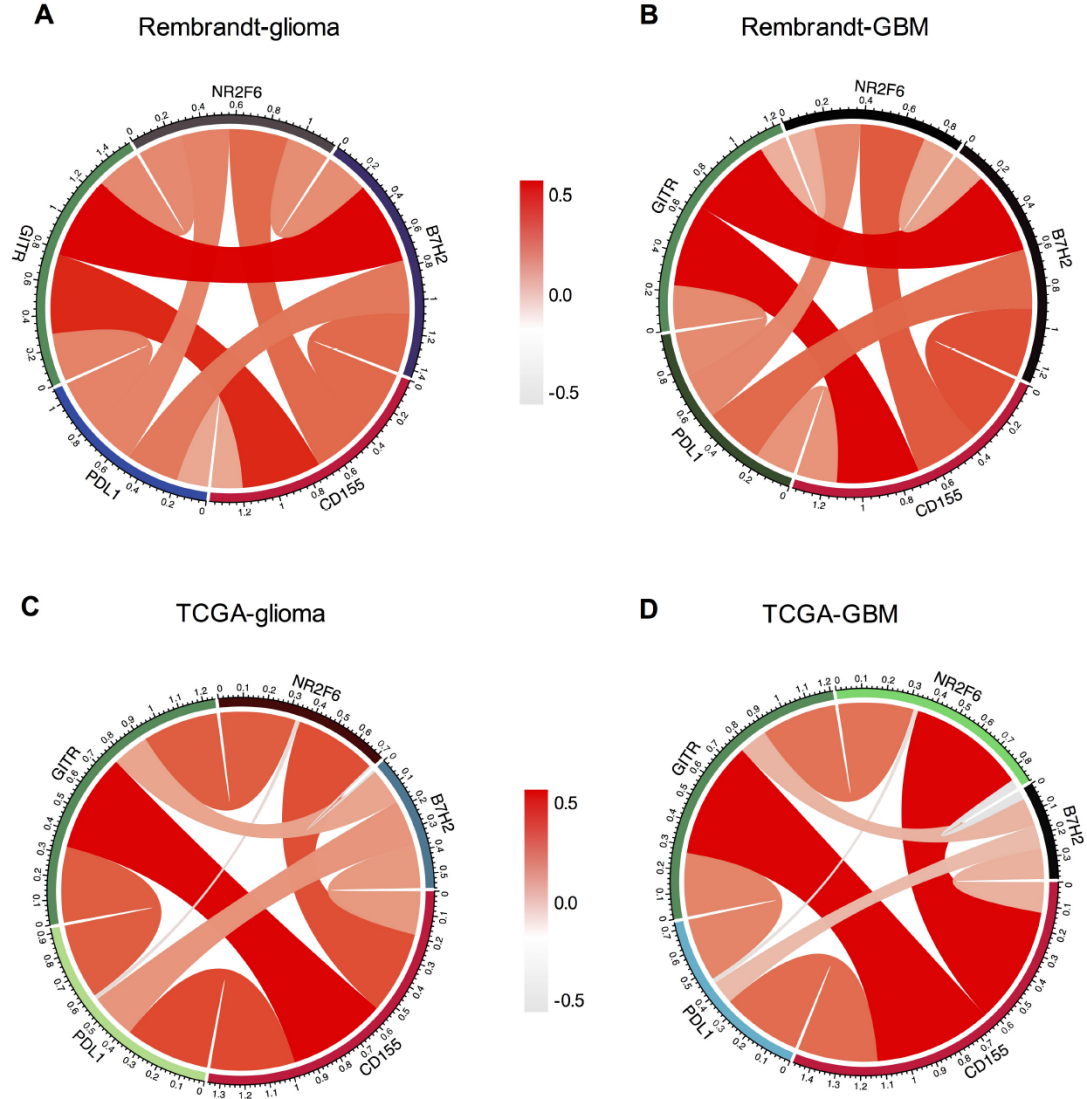
**Association between CD155 and immune checkpoint markers in glioma.** Correlation between CD155 and immune checkpoint markers (PD-L1, PD-L2, NR2F6, and B7-H2) in gliomas (**A**) and GBM (**B**) in Rembrandt dataset were subjected to the analysis. Correlation between CD155 and immune checkpoint markers (PD-L1, PD-L2, NR2F6, and B7-H2) in gliomas (**C**) and GBM (**D**) in TCGA were also included.

Recent study has demonstrated the enhanced antitumor effect of contemporary immune checkpoints in CD155–/– mice, suggesting the potential clinical combination of these immune checkpoints blockade and CD155 inhibition [8]. Thus, we analyzed the relationship between CD155 and other immune checkpoint markers that have been examined in clinical trials or clinical situations. Pearson correlation analysis was performed using both Rembrandt and TCGA datasets. After screening several immune chekpoints including PD-1, PD-L1, PD-L2, CTLA-4, TIM-3, B7-H2, NR2F6 and GITR, we found that CD155 was positively associated with PD-L1, B7-H2, NR2F6 and GITR as shown by Circos plots. Similar results were found in all grades glioma and GBM samples in Rembrandt and TCGA datasets (Figure 6), indicating the possible synergistic effects of CD155 with these checkpoint members.

**Figure 7:**
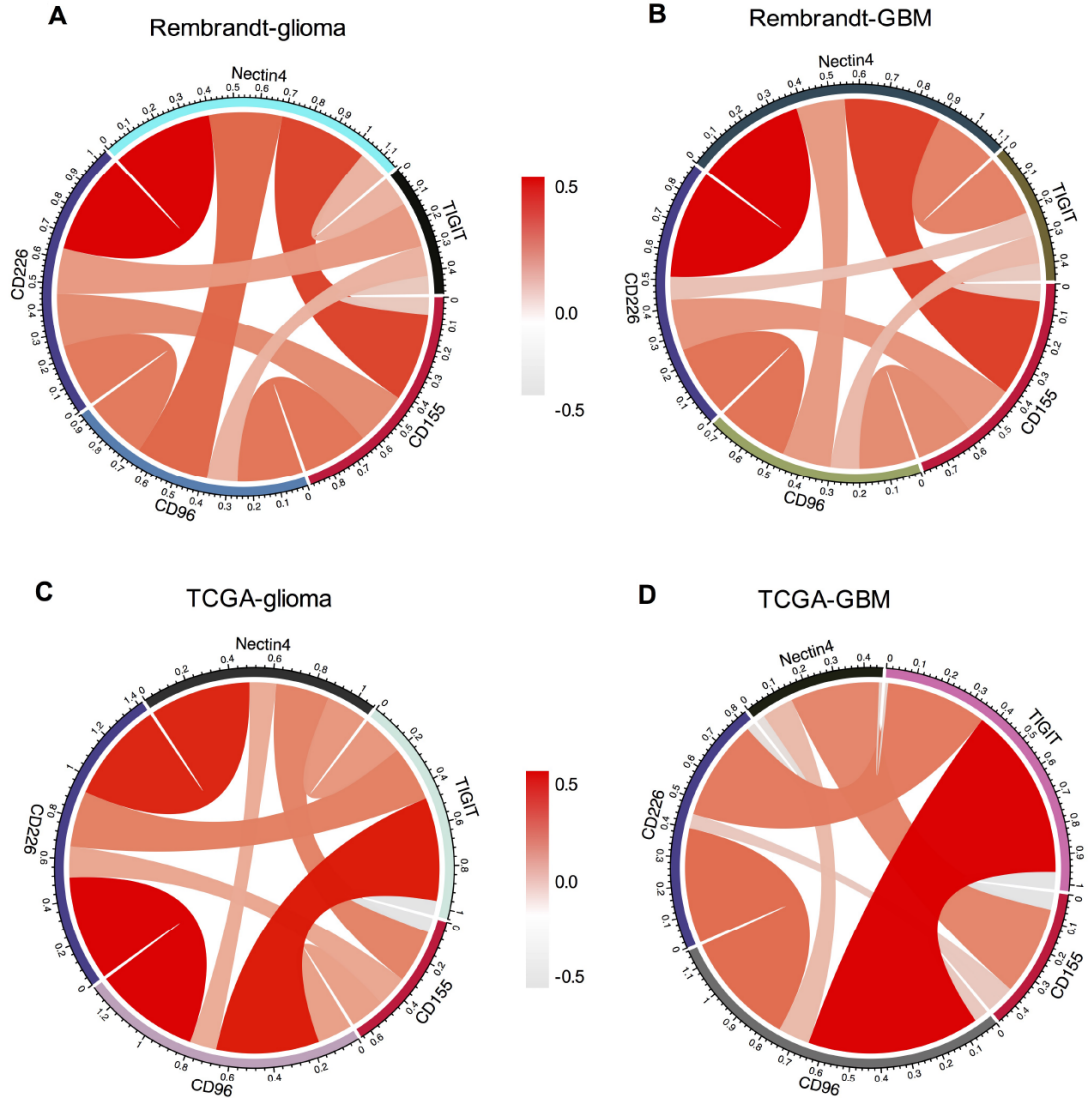
**Association between CD155 and immune checkpoint markers in glioma.** The correlations between CD155 and immunoglobulin family members including TIGIT, CD226, CD96 based on Rembrandt (**A** and **B**) and TCGA (**C** and **D**) datasets were presented. Ligands Nectin4 was also included in the analysis.

As an immunoglobulin-like adhesion molecule, CD155 is the ligands for immunoglobulin (Ig) superfamily members including TIGIT, CD96 and CD226 [16, 17, 21]. Members in Ig family are gaining increasing attention as immune checkpoint receptor targets for cancer immunotherapy [11, 17, 20]. Therefore, we detected the relationship among CD155, TIGIT, CD226, CD96, CD112, and Nectin4. Recently, CD155 and CD112 have been identified as novel immune checkpoints [21]. We analyzed the expression of CD112 in glioma samples by immunohistochemical staining assay. CD112 can be detected in CD155-positive glioma samples (Supplementary Figure 2). Pearson correlation analysis didn’t find significant correlation between CD155 and CD112 in glioma (r = 0.089, p = 0.052727) and GBM (r = 0.125, p = 0.059775) in Rembrandt dataset. There was a positive correlation between CD155 and CD112 in glioma (r = 0.590, p = 1.1734E-66) and GBM (r = 0.627, p = 9.3073E-20) in TCGA dataset. The survival analysis based on high/low CD155 expression and high/low CD112 expression showed that patients with high CD155 and CD112 expressions had the worst survival in both Rembrandt and TCGA datasets (Supplementary Figure 3). CD155 showed strong positive concordance with CD96, CD226, and Nectin4 in all gliomas and GBM samples from Rembrandt dataset (Figure 7A, 7B). Subsequent analysis in TCGA dataset indicated that CD155 was positive associated with CD96, CD226, and Nectin4 in glioma (Figure 7C) and GBM samples (Figure 7D). No significant correlation between CD155 and TIGIT were detected in both datasets. Detailed information of r and adjusted p values between CD155 and these immune-related markers were provided in Supplementary Table 4.

### CD155 predicts worse survival in glioma patients

**Figure 8:**
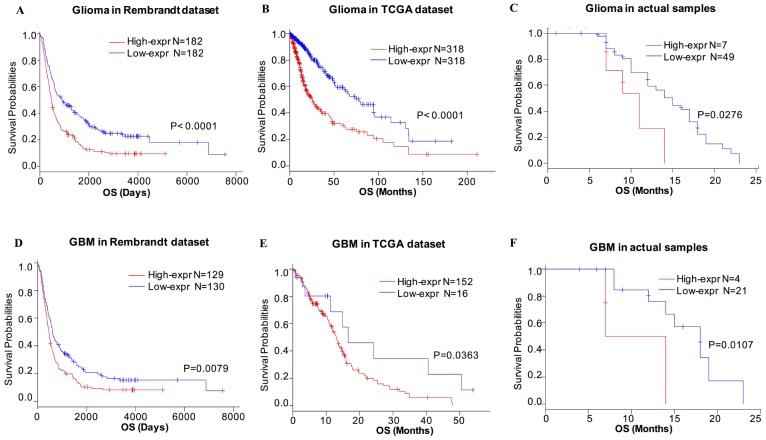
**Survival analysis of glioma based on CD155 expression.** Higher CD155 expression is associated with worse overall survival (OS) in patients with glioma (**A**–**C**) and GBM (**D**–**F**) based on data from Rembrandt dataset, TCGA dataset, and the follow-up data from 56 glioma samples from Xiangya Hospital.

Based on our findings, CD155 was expressed at higher levels with higher grades of glioma, indicating a malignant biological property of this marker, based on Rembrandt and TCGA datasets. Therefore, we evaluated the prognostic value of CD155 based on these two glioma datasets to determine its effect on patient survival. Figure 8 depicts the Kaplan–Meier curve of the overall survival (OS) of patients with gliomas. As shown, higher CD155 expression predicted worse overall survival for glioma in Rembrandt dataset (Figure 8A). Similarly, a strong association was observed between higher expression of CD155 and shorter patient OS for glioma and GBM patients in TCGA dataset (Figure 8B). The follow-up data from 56 glioma samples also suggested that higher CD155 expression was correlated with a worse overall survival (Figure 8C). Highly consistent results were found in GBM samples (Figure 8D–8F). These findings indicated that CD155 is a negative prognostic indicator in glioma and GBM patients.

## DISCUSSION

As one of the most promising approaches to activate antitumor immune response, immune checkpoint inhibitors have shown remarkable success for various cancer treatments [6, 22]. They also have demonstrated promising benefits for glioma patients based on several studies [6]. However, not all cancer patients benefit from these therapies. Current immune checkpoint blockade can also cause immune-related adverse events, which should be considered [6, 23]. These findings encouraged us to identify a new alterative checkpoint target that could result in enhanced therapeutic benefits for cancer treatment.

Recent studies demonstrated that CD155 plays an important role in anti-tumor immune responses [9]. Hepatocellular carcinoma patients with up-regulated CD155 expression within tumor are strongly associated with worse prognosis, the presence of anti-CD155 antibody can significantly increases lysis of hepatoma cell line HepG2 by NK cells [24]. Targeting host CD155 could thus act as a promising approach for combination with current immunotherapies. In our study, we explored the genetic and clinical characteristics of CD155 expression based on 1173 glioma samples. Higher CD155 expression was observed with higher WHO grade gliomas. Moreover, CD155 was found to predict worse survival for glioma and GBM patients. These results indicated a malignant biological property for CD155 in glioma. Emerging data has demonstrated a critical role for CD155 in tumor immunology. To further demonstrate the relationship between CD155 expression and immune responses, we explored the relationship between CD155 and immune metagenes, and found that CD155 expression was particularly associated with T-cell and macrophage-related, rather than B cell linage-related immune responses. These findings indicate that CD155 is a negative prognostic factor for glioma and plays an important role in the immune response.

CD155 is abundant in various human tumors [9, 11]. As the common ligand for co-inhibitory receptors CD96, TIGIT and co-stimulatory receptor CD226, CD155 seems to play a dual role in oncoimmunity. TIGIT and CD226 are well-studied receptors that bind CD155 and CD112 (nectin-2). CD96 shares its ligand CD155 with CD226 and TIGIT, but also binds CD111 (nectin-1) [17]. Studies found CD155 showed high affinity for binding to TIGIT, caused more inhibition and exhaustion of TIGIT^+^ NK cells [25]. However, the relationship between CD155 and these receptors in gliomas is unknown. We observed that CD155 was positively associated with CD96, and CD226 in both datasets. This is consistent with network analysis derived from the BioPAX that demonstrated genes including CD96 and CD226 can form complex with CD155. We also examined the relationship between CD155 and CD112, compared to the tight relationship in TCGA dataset, the correlation between CD155 and CD112 was not significant in Rembrandt dataset. Further survival analysis showed that patients with high CD155 and CD112 expressions had the worst survival compared with other groups, suggesting the possible coordinated effects of them.

Different clinical trials of checkpoint inhibitors for gliomas, and especially GBM, are ongoing [6]. Immunotherapies that target combined checkpoint inhibitory pathways have demonstrated profound clinical benefits compared to that with monotherapy treatments [26, 27]. Specifically, compared to checkpoint monotherapy, combination approaches were reported to be more effective and associated with significantly longer progression-free survival [26]. CD155 loss enhances tumor suppression of multiple immune checkpoint blockades [8]. Our results showed that CD155 is tightly associated with the checkpoint proteins PD-L1, B7-H2, NR2F6 and GITR, indicating the potential synergistic effects of these markers.

In summary, these findings would broaden our knowledge of the expression and clinical characteristics of CD155 in gliomas. Moreover, CD155 is a promising immunotherapy target that positively collaborates with other checkpoint proteins in glioma. Future research is needed to further explore CD155-targeted antitumor immunotherapeutics combined with multi-checkpoint blockade for glioma treatment.

## MATERIALS AND METHODS

### Sample and data collection

#### Glioma specimens and related data collection from Xiangya Hospital

We identified formalin-fixed and paraffin-embedded glioma specimens of 58 adult patients who underwent neurosurgical resection of gliomas at the Department of Neurosurgery, Xiangya Hospital from the tissue specimen database of the Institute of Neurology, Xiangya Hospital, Central South University. Three additional normal brain tissues were included. Historical diagnosis of glioma was performed according to WHO classification. A total of 61 specimens were analyzed, including three normal brain tissues, 20 tumor specimens of glioma grade I-II, 12 tumor specimens of glioma grade III, and 26 tumor specimens of glioma grade IV. Two patients were lost to follow-up because of death, including one patient of grade III glioma and one patients of grade IV glioma. Therefore, we included survival analysis based on CD155 expression of 56 actual samples. The ethics committee of the Xiangya Hospital of Central South University approved the study (No. 2017121019).

#### Data from Rembrandt and TCGA datasets

The clinical and biospecimen data from 474 glioma patients in the Rembrandt brain cancer dataset (https://www.ncbi.nlm.nih.gov/geo/query/acc.cgi?acc=GSE108475) were included in the analysis. Collected from 14 contributing institutions, the Rembrandt dataset includes gene expression and copy number changes alongside clinical outcomes from clinical trials involving patients suffering from gliomas. To maintain consistency, we also validated our findings using the TCGA dataset. The clinicopathological characteristics and RNA-seq data from 699 glioma samples of all grades, ranging from WHO grade II to grade IV, were analyzed in our study (http://cancergenome.nih.gov).

### Immunohistochemical staining and analysis

For immunohistochemical staining, the slides were deparaffinized in 60°c incubator for 3 hours, incubated in xylene, 100% ethanol, 95% ethanol, 80% ethanol, 70% ethanol, and dH_2_O in turn. The citrate buffer (PH=6.0) was used for antigen unmasking. After blocking, slides were treated with anti-CD155 (rabbit, Cell Signaling 81254, 1:200) in Antibody Diluent (Cell Signaling, 8112L). Goat anti-Rabbit IgG (H+L) Secondary Antibody, HRP was used (Invitrogen 31460, 1:1000). The images of immunohistochemical staining were acquired by an Olympus microscope.

### Statistical analysis

R language was mainly used to perform statistical analysis [28]. Gene expression profiling data were log-transformed for further analysis. A Student’s t test was performed to evaluate CD155 expression differences between grades and subtypes of gliomas. The GSVA package of R language was employed to determine the enrichment status of inflammatory response-associated metagenes [29]. Pearson correlation and correlograms were performed using the “circlize” package [30] and “corrgram” package, respectively, and ROC curves were derived using the “pROC” package [31]. The prognostic value of CD155 was investigated by Kaplan–Meier analysis using R language (survival package) [32]. The Kaplan–Meier curves were constructed based on the mean of the expression of CD155 to stratify patients. A heatmap was generated by clustering (using the R package “pheatmap”) [33] based on p-values < 0·05 between two groups. All statistical tests were two-sided and p < 0.05 was considered a significant difference.

## Supplementary Material

Supplementary Figures

Supplementary Tables

Supplementary Table 1
