# Supplementary material for: Large-scale analysis reveals the specific clinical and immune features of CD155 in glioma
Source: Aging (Albany NY). 2019 Aug 4;11(15):5463–82. doi: 10.18632/aging.102131 (PMC6710042; doi:10.18632/aging.102131)
Supplement: Supplementary Figures [file aging-11-102131-s003.pdf]

SUPPLEMENTARY FIGURES

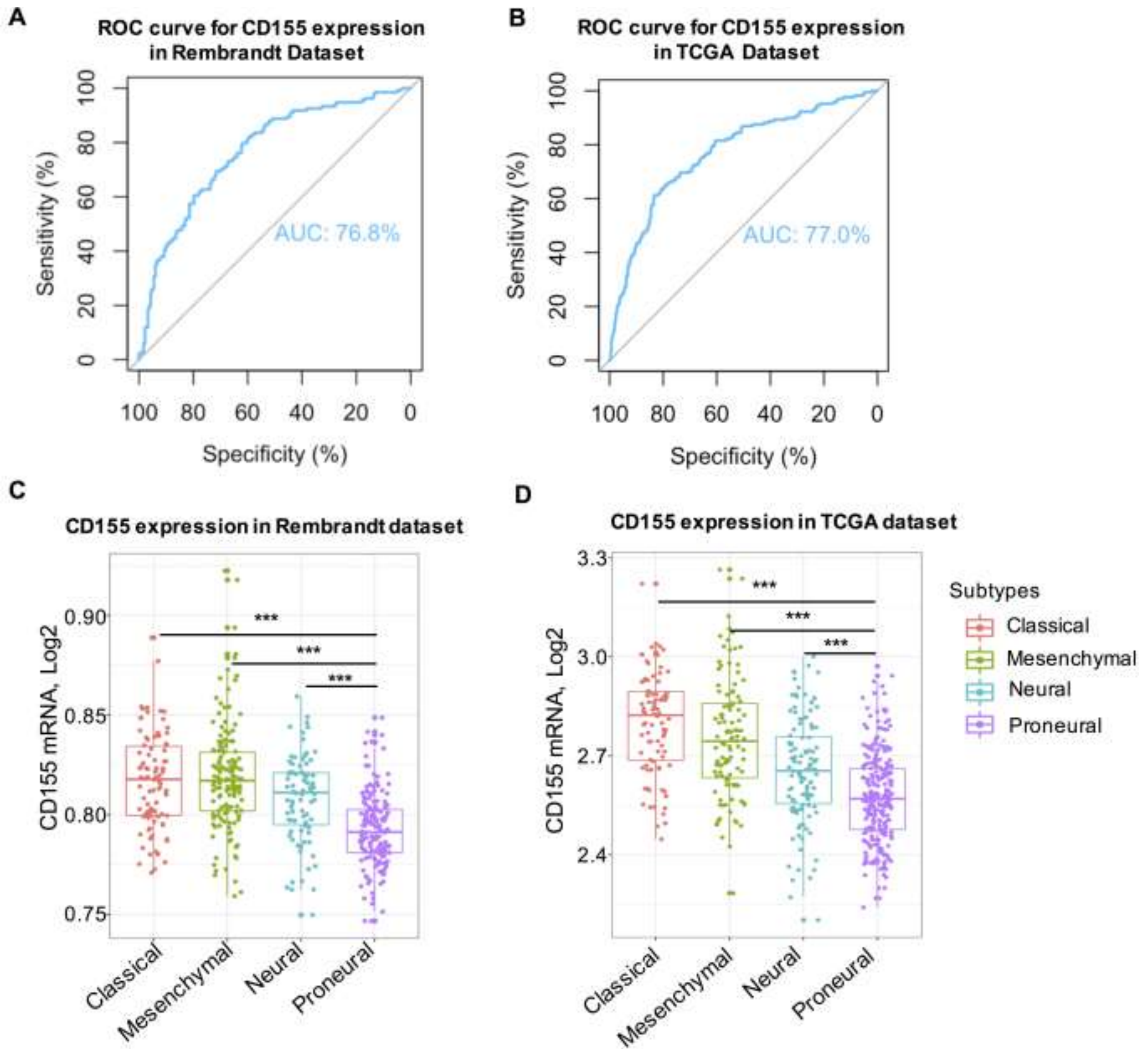

Supplementary Figure 1. Relationship between CD155 expression and glioma molecular subtypes. ROC curve analysis revealed the predictive value of CD155 expression in GBM based on both datasets (**A** and **B**). CD155 expression pattern in different molecular subtypes of gliomas based on Rembrandt (**C**) and TCGA (**D**) datasets.

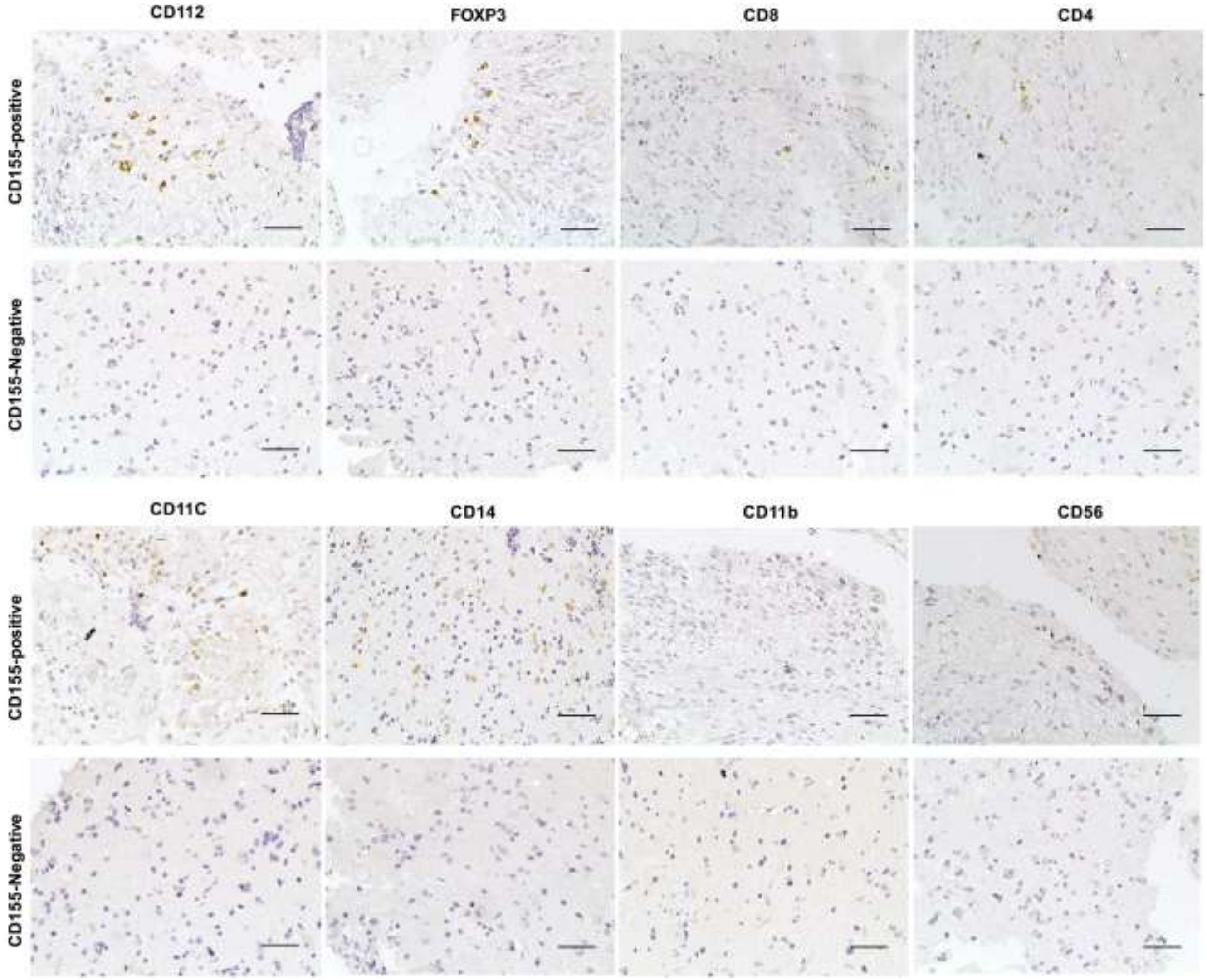

Supplementary Figure 2. Expression of CD112, CD11b, CD14, CD56, CD11c, FOXP3, CD8 and CD4 in CD155-positive and CD155-negative glioma samples. Scale bars, 100 mm.

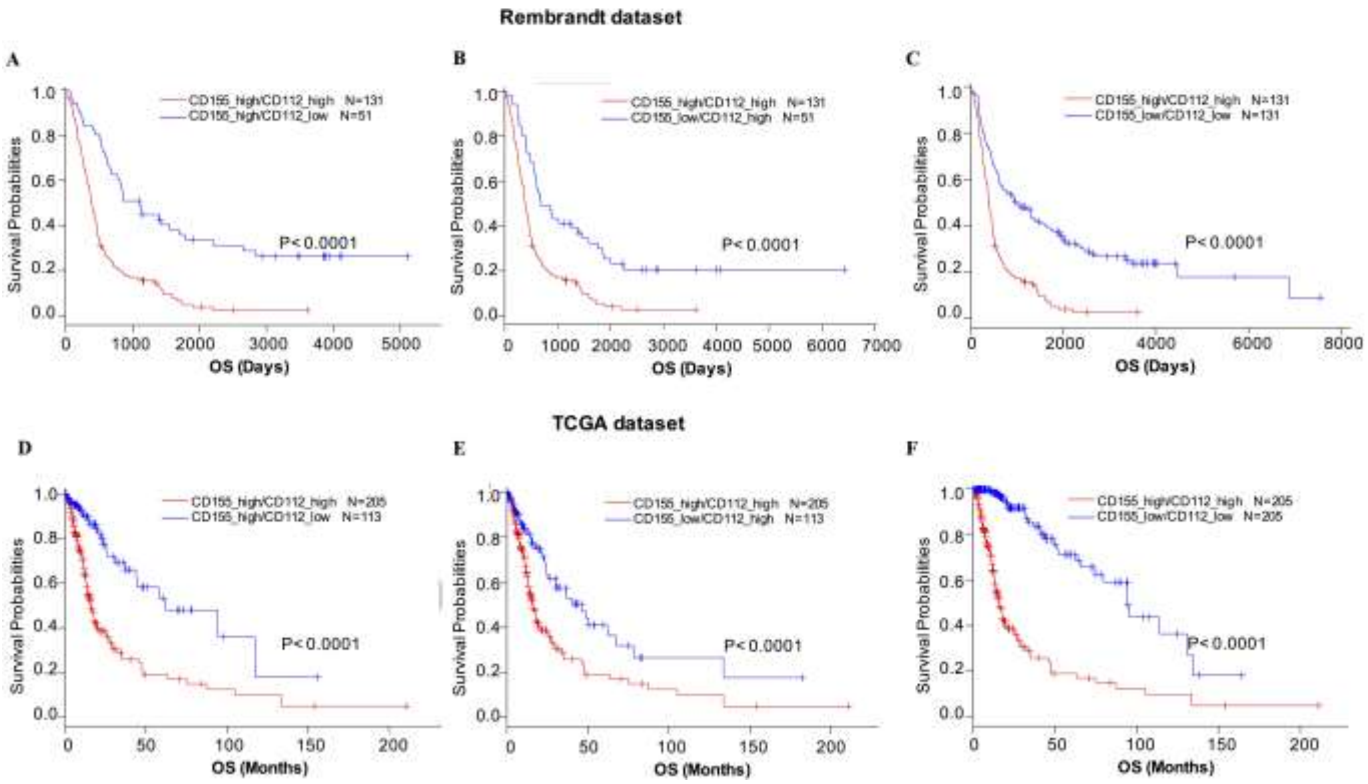

Supplementary Figure 3. Survival analysis based on high/low CD155 expression and high/low D112 expression in Rembrandt (**A**) and TCGA (**B**) datasets.
