# Supplementary material for: Large-scale analysis reveals the specific clinical and immune features of CD155 in glioma
Source: Aging (Albany NY). 2019 Aug 4;11(15):5463–82. doi: 10.18632/aging.102131 (PMC6710042; doi:10.18632/aging.102131)
Supplement: Supplementary Tables [file aging-11-102131-s002.pdf]

SUPPLEMENTARY TABLES

Please browse Full Text version to see the data of Supplementary Table 1:

Supplementary Table 1. List of immune responses related genes which significantly correlated with CD155 expression in Rembrandt and TCGA datasets.

Supplementary Table 2. A detailed list of the 104 genes from seven metagenes.

Gene	Metagene
C1QB	HCK
C1QA	HCK
AIF1	HCK
LST1	HCK
DOCK2	HCK
LAPTM5	HCK
TYROBP	HCK
MS4A4A	HCK
MS4A6A	HCK
CD163	HCK
ITGB2	HCK
SLC7A7	HCK
LAIR1	HCK
HCK	HCK
TFEC	HCK
IFI30	HCK
MNDA	HCK
FCER1G	HCK
RNASE6	HCK
SLCO2B1	HCK
CCR1	HCK
IGSF8	IgG
ISLR2	IgG
IGSF21	IgG
IGSF1	IgG
IGSF22	IgG
IGDCC3	IgG
IGHD	IgG
IGSF11	IgG
IGSF5	IgG
IGSF6	IgG
IFIT1	Interferon
IFIT3	Interferon
IFI44L	Interferon
OAS3	Interferon
MX1	Interferon
RSAD2	Interferon

IFI44	Interferon
OAS2	Interferon
OAS1	Interferon
CD2	LCK
GZMK	LCK
GZMA	LCK
CD3D	LCK
CD53	LCK
LCK	LCK
ARHGAP15	LCK
CCL5	LCK
GMFG	LCK
SELL	LCK
STAT4	LCK
SAMSN1	LCK
RAC2	LCK
HCLS1	LCK
CCR7	LCK
PIK3CD	LCK
CORO1A	LCK
CD48	LCK
IL2RG	LCK
SH2D1A	LCK
SLAMF1	LCK
IL7R	LCK
INPP5D	LCK
KLRK1	LCK
FGL2	LCK
IRF8	LCK
SELPLG	LCK
IL10RA	LCK
SLA	LCK
CCR2	LCK
CSF2RB	LCK
HLA-E	MHC_I
HLA-H	MHC_I
HLA-B	MHC_I
HLA-J	MHC_I
HLA-F	MHC_I
HLA-G	MHC_I
HLA-A	MHC_I
HLA-C	MHC_I
HLA-L	MHC_I
HLA-DRB1	MHC_II
HLA-DRB5	MHC_II
HLA-DRB3	MHC_II
HLA-DPA1	MHC_II
HLA-DRA	MHC_II

HLA-DQA1	MHC_II
HLA-DQA2	MHC_II
HLA-DMA	MHC_II
HLA-DOA	MHC_II
HLA-DRB4	MHC_II
HLA-DMB	MHC_II
HLA-DQB1	MHC_II
HLA-DPB1	MHC_II
HLA-DQB2	MHC_II
CD74	MHC_II
PTPRC	MHC_II
HLA-DOB	MHC_II
HLA-DPB2	MHC_II
TAP1	STAT1
STAT1	STAT1
CXCL10	STAT1
CXCL11	STAT1
GBP1	STAT1
CXCL9	STAT1

Supplementary Table 3. A detailed list of specific biomarkers of eight immune cell types.

inflammatory cell type	biomarker
Macrophages	CD14
Macrophages	HLADRA
Macrophages	CD312
Macrophages	CD115
Macrophages	CD163
Macrophages	CD204
Macrophages	CD301
Macrophages	CD206
Neutrophils	CD11b
Neutrophils	CD16
Neutrophils	CD66b
Neutrophils	ELANE
MDSCs	CD14
MDSCs	CD16
MDSCs	CD33
MDSCs	ARG1
CD8T	CD3E
CD8T	CD8A
NK	CD16
NK	CD56
Tregs	CD3E
Tregs	CD4
Tregs	CD25
Tregs	FOXP3
CD4T	CD3E

CD4T	CD4
DC	CD11b
DC	CD11c

Supplementary Table 4. Detailed information of correlations between CD155 and other immune markers in Rembrandt and TCGA datasets.

Correlations between CD155 and other immune markers in Rembrandt dataset

	Glioma		GBM	
**markers**	**r**	**p**	**r**	**p**
PD-L1	0.190	0.000030	0.206	0.001854
GITR	0.484	2.7549E-29	0.427	1.8349E-11
NR2F6	0.355	1.3494E-15	0.340	1.5539E-7
B7-H2	0.363	3.0646E-16	0.363	1.7472E-8
CD96	0.255	1.6272E-8	0.198	0.002667
CD226	0.206	0.000006	0.183	0.005814
Nectin4	0.353	2.188E-15	0.338	1.8366E-7
TIGIT	0.073	0.113151	0.055	0.408430
CD112	0.089	0.052727	0.125	0.059775

Correlations between CD155 and other immune markers in TCGA dataset

	Glioma		GBM	
**markers**	**r**	**p**	**r**	**p**
PD-L1	0.367	8.9678E-24	0.343	0.000005
GITR	0.428	1.8544E-32	0.505	3.0394E-12
NR2F6	0.360	8.0572E-23	0.504	3.2147E-12
B7-H2	0.194	2.3076E-7	0.127	0.101887
CD96	0.173	0.000004	0.048	0.533583
CD226	0.146	0.000103	0.054	0.483812
Nectin4	0.258	4.4827E-12	0.313	0.000037
TIGIT	-0.074	0.051879	-0.062	0.427740
CD112	0.590	1.1734E-66	0.627	9.3073E-20
