# Supplementary material for: Large-scale analysis reveals the specific clinical and immune features of CD155 in glioma
Source: Aging (Albany NY). 2019 Aug 4;11(15):5463–82. doi: 10.18632/aging.102131 (PMC6710042; doi:10.18632/aging.102131)
Supplement: Supplementary Table 1 [file aging-11-102131-s001.docx]

Supplementary Table 1. List of genes in Rembrandt and TCGA datasets that are involved in immune responses in humans and significantly correlated with CD155 expression.

| Rembrandt |  |  |  |  | TCGA |  |  |  |  |
| --- | --- | --- | --- | --- | --- | --- | --- | --- | --- |
| gene | cor | t.value | p.value | FDR | gene | cor | t.value | p.value | FDR |
| DEFB118 | 0.61525313 | 16.9737014 | 8.25E-51 | 5.40E-47 | SBNO2 | 0.62888918 | 21.3546733 | 3.14E-78 | 1.06E-74 |
| MUC5AC | 0.60628829 | 16.580928 | 5.21E-49 | 1.46E-45 | PRKACA | 0.61501426 | 20.5916464 | 5.85E-74 | 1.31E-70 |
| SMPDL3B | 0.59314751 | 16.0231094 | 1.80E-46 | 2.52E-43 | BCL3 | 0.59159302 | 19.3721062 | 3.25E-67 | 3.86E-64 |
| CIITA | 0.58842088 | 15.8273855 | 1.38E-45 | 1.59E-42 | GPI | 0.58330236 | 18.9590978 | 5.87E-65 | 3.90E-62 |
| KIR2DS1 | 0.56087436 | 14.7339022 | 1.04E-40 | 4.34E-38 | CD93 | 0.57499549 | 18.5542927 | 9.25E-63 | 4.80E-60 |
| CNR2 | 0.55763862 | 14.6103781 | 3.64E-40 | 1.25E-37 | SLC2A3 | 0.56764338 | 18.2031806 | 7.24E-61 | 2.93E-58 |
| CD244 | 0.55505432 | 14.5124178 | 9.78E-40 | 3.15E-37 | RUNX1 | 0.5560276 | 17.6614398 | 5.69E-58 | 1.77E-55 |
| C8B | 0.55122726 | 14.3684626 | 4.17E-39 | 1.12E-36 | CD276 | 0.54990462 | 17.3819698 | 1.72E-56 | 5.12E-54 |
| PTK6 | 0.5506567 | 14.3471135 | 5.16E-39 | 1.35E-36 | IQGAP1 | 0.54870378 | 17.3276359 | 3.34E-56 | 9.64E-54 |
| NCR1 | 0.54848286 | 14.2660359 | 1.17E-38 | 2.89E-36 | PLAUR | 0.54832633 | 17.3105894 | 4.10E-56 | 1.15E-53 |
| DHX9 | -0.5441984 | -14.107445 | 5.69E-38 | 1.20E-35 | KDELR1 | 0.54357279 | 17.0971915 | 5.45E-55 | 1.38E-52 |
| SERPINB3 | 0.53973196 | 13.9437869 | 2.90E-37 | 5.27E-35 | ULBP3 | 0.5388553 | 16.8877212 | 6.81E-54 | 1.56E-51 |
| CD1B | 0.53213979 | 13.6693873 | 4.38E-36 | 6.10E-34 | RFTN2 | -0.5349155 | -16.714499 | 5.44E-53 | 1.12E-50 |
| IKBKB | -0.5314398 | -13.644321 | 5.61E-36 | 7.69E-34 | CAMK2D | 0.53459249 | 16.7003672 | 6.44E-53 | 1.30E-50 |
| TNK1 | 0.52731445 | 13.4973917 | 2.37E-35 | 2.97E-33 | RAB3D | 0.53385723 | 16.6682333 | 9.46E-53 | 1.86E-50 |
| RAF1 | -0.5251507 | -13.420856 | 5.02E-35 | 5.79E-33 | ULBP2 | 0.52835933 | 16.4296158 | 1.63E-51 | 2.86E-49 |
| IL17A | 0.52461705 | 13.4020361 | 6.03E-35 | 6.88E-33 | SOCS3 | 0.52725166 | 16.3818913 | 2.87E-51 | 4.68E-49 |
| PDCD1LG2 | 0.52238724 | 13.3236313 | 1.30E-34 | 1.35E-32 | MYO1C | 0.52643897 | 16.3469495 | 4.35E-51 | 7.03E-49 |
| PIGR | 0.52214054 | 13.3149798 | 1.41E-34 | 1.46E-32 | CLCF1 | 0.51960327 | 16.0554742 | 1.36E-49 | 1.95E-47 |
| CCL11 | 0.52194858 | 13.3082514 | 1.51E-34 | 1.53E-32 | TXNDC5 | 0.5130789 | 15.7812157 | 3.38E-48 | 4.33E-46 |
| CPNE3 | -0.5215433 | -13.294055 | 1.73E-34 | 1.72E-32 | LIMK1 | 0.50916909 | 15.6186509 | 2.25E-47 | 2.77E-45 |
| DEFB125 | 0.51807171 | 13.1729515 | 5.60E-34 | 5.04E-32 | ITGB1 | 0.5072416 | 15.5389905 | 5.66E-47 | 6.54E-45 |
| LY9 | 0.51797259 | 13.1695071 | 5.79E-34 | 5.19E-32 | FKBP1A | 0.50199698 | 15.3238177 | 6.79E-46 | 7.31E-44 |
| EPX | 0.51488557 | 13.0625895 | 1.63E-33 | 1.35E-31 | PLAU | 0.5013577 | 15.2977456 | 9.17E-46 | 9.76E-44 |
| KRT1 | 0.51176382 | 12.9551723 | 4.59E-33 | 3.38E-31 | LIF | 0.50103385 | 15.2845504 | 1.07E-45 | 1.12E-43 |
| KLK7 | 0.51045072 | 12.910198 | 7.07E-33 | 5.02E-31 | ANXA2 | 0.50030226 | 15.2547739 | 1.50E-45 | 1.57E-43 |
| SIN3A | -0.5080602 | -12.828634 | 1.55E-32 | 1.04E-30 | JAG1 | 0.49523325 | 15.0496481 | 1.57E-44 | 1.54E-42 |
| DYNLT1 | -0.5073797 | -12.80549 | 1.93E-32 | 1.27E-30 | NCAM1 | -0.4941063 | -15.004323 | 2.64E-44 | 2.51E-42 |
| CD1A | 0.50716204 | 12.798093 | 2.07E-32 | 1.35E-30 | IL1RAP | 0.49274371 | 14.9496536 | 4.91E-44 | 4.60E-42 |
| CD80 | 0.50441224 | 12.7049359 | 5.04E-32 | 3.05E-30 | CD97 | 0.49253148 | 14.9411518 | 5.41E-44 | 5.04E-42 |
| CD19 | 0.50253648 | 12.6416869 | 9.20E-32 | 5.34E-30 | PYGL | 0.49138928 | 14.8954559 | 9.09E-44 | 8.21E-42 |
| CLEC4D | 0.50241815 | 12.637705 | 9.55E-32 | 5.51E-30 | ALDOC | -0.4894267 | -14.817177 | 2.21E-43 | 1.92E-41 |
| SOS1 | -0.5000785 | -12.559166 | 2.01E-31 | 1.10E-29 | CANT1 | 0.48833055 | 14.7735832 | 3.61E-43 | 3.08E-41 |
| CCL25 | 0.49338135 | 12.336369 | 1.65E-30 | 7.60E-29 | SHC1 | 0.4857972 | 14.6731856 | 1.12E-42 | 9.04E-41 |
| CTLA4 | 0.49316812 | 12.3293234 | 1.76E-30 | 8.09E-29 | RAB5B | -0.4852744 | -14.652527 | 1.42E-42 | 1.14E-40 |
| SFTPA2 | 0.49111397 | 12.2616009 | 3.32E-30 | 1.44E-28 | IRAK2 | 0.48516624 | 14.6482561 | 1.49E-42 | 1.18E-40 |
| TNFSF11 | 0.48958504 | 12.211369 | 5.31E-30 | 2.25E-28 | FZD5 | 0.48505319 | 14.6437924 | 1.56E-42 | 1.24E-40 |
| ZC3HAV1 | -0.489275 | -12.201202 | 5.84E-30 | 2.46E-28 | IFNGR2 | 0.48443313 | 14.6193278 | 2.06E-42 | 1.62E-40 |
| CTSG | 0.48794181 | 12.1575475 | 8.78E-30 | 3.60E-28 | RELB | 0.48280225 | 14.5551182 | 4.23E-42 | 3.20E-40 |
| CD3G | 0.4874985 | 12.1430564 | 1.01E-29 | 4.08E-28 | FUCA2 | 0.48277307 | 14.5539712 | 4.28E-42 | 3.23E-40 |
| CCL13 | 0.48722101 | 12.1339922 | 1.09E-29 | 4.42E-28 | RPS6KA3 | 0.47924163 | 14.4156171 | 2.01E-41 | 1.42E-39 |
| YES1 | -0.4850349 | -12.062751 | 2.12E-29 | 8.28E-28 | TRPM4 | 0.47718319 | 14.3353921 | 4.92E-41 | 3.25E-39 |
| SERPINB10 | 0.48428762 | 12.0384652 | 2.66E-29 | 1.01E-27 | STBD1 | 0.47456216 | 14.2336829 | 1.52E-40 | 9.59E-39 |
| ZP4 | 0.48248597 | 11.9800574 | 4.57E-29 | 1.69E-27 | ICAM1 | 0.4722231 | 14.1433281 | 4.14E-40 | 2.50E-38 |
| FGA | 0.48126276 | 11.9405152 | 6.58E-29 | 2.39E-27 | GALNS | 0.46793812 | 13.9787991 | 2.53E-39 | 1.40E-37 |
| VIPR1 | 0.48011042 | 11.9033468 | 9.28E-29 | 3.28E-27 | PLA2G6 | -0.4630172 | -13.791408 | 1.96E-38 | 1.02E-36 |
| ILF2 | -0.4788662 | -11.863306 | 1.34E-28 | 4.61E-27 | RAB27A | 0.46267328 | 13.7783726 | 2.26E-38 | 1.16E-36 |
| RELA | -0.478249 | -11.843478 | 1.61E-28 | 5.48E-27 | IMPDH1 | 0.460766 | 13.706225 | 4.96E-38 | 2.44E-36 |
| DERA | -0.4776028 | -11.822741 | 1.95E-28 | 6.51E-27 | CLEC5A | 0.46032799 | 13.6896903 | 5.93E-38 | 2.88E-36 |
| TSPAN6 | -0.4771089 | -11.806908 | 2.25E-28 | 7.47E-27 | GUSB | 0.45886061 | 13.6343895 | 1.08E-37 | 5.08E-36 |
| RORC | 0.47560293 | 11.7587244 | 3.51E-28 | 1.13E-26 | SWAP70 | 0.45797464 | 13.6010687 | 1.55E-37 | 7.16E-36 |
| PRKCQ | 0.47477885 | 11.7324139 | 4.46E-28 | 1.42E-26 | ADORA2B | 0.45593783 | 13.5246593 | 3.53E-37 | 1.59E-35 |
| KIR2DL3 | 0.47470127 | 11.7299392 | 4.56E-28 | 1.45E-26 | THBS1 | 0.45403074 | 13.4533597 | 7.60E-37 | 3.35E-35 |
| ZBTB1 | -0.4734459 | -11.689941 | 6.58E-28 | 2.02E-26 | DDOST | 0.44829916 | 13.2404685 | 7.40E-36 | 3.03E-34 |
| CCR4 | 0.4706655 | 11.6016799 | 1.47E-27 | 4.24E-26 | ACTR3 | 0.44827314 | 13.2395067 | 7.47E-36 | 3.06E-34 |
| CCL16 | 0.47052557 | 11.5972498 | 1.53E-27 | 4.40E-26 | CTSA | 0.44717791 | 13.1990621 | 1.15E-35 | 4.58E-34 |
| JAK3 | 0.47048177 | 11.5958634 | 1.55E-27 | 4.45E-26 | VAV3 | 0.44699439 | 13.1922924 | 1.23E-35 | 4.90E-34 |
| PRSS2 | 0.46857937 | 11.5357497 | 2.68E-27 | 7.41E-26 | RAG1 | 0.44655265 | 13.176006 | 1.47E-35 | 5.80E-34 |
| NCK1 | -0.4677985 | -11.511133 | 3.35E-27 | 9.03E-26 | TNFAIP6 | 0.44630877 | 13.1670193 | 1.61E-35 | 6.36E-34 |
| IL19 | 0.46713176 | 11.4901444 | 4.05E-27 | 1.08E-25 | HEXB | 0.44574614 | 13.1463018 | 2.01E-35 | 7.83E-34 |
| LBP | 0.46537007 | 11.434804 | 6.67E-27 | 1.72E-25 | CEBPB | 0.43880299 | 12.8922174 | 2.92E-34 | 1.05E-32 |
| IL2RA | 0.4649898 | 11.4228813 | 7.43E-27 | 1.90E-25 | CD300E | 0.43816202 | 12.8689072 | 3.73E-34 | 1.32E-32 |
| PGLYRP4 | 0.46343001 | 11.3740612 | 1.15E-26 | 2.87E-25 | RNF19B | 0.43703431 | 12.8279539 | 5.72E-34 | 2.00E-32 |
| CAMK2A | 0.4618216 | 11.3238602 | 1.81E-26 | 4.36E-25 | SUSD2 | 0.43688334 | 12.822477 | 6.06E-34 | 2.11E-32 |
| DEFA5 | 0.45984342 | 11.2623126 | 3.15E-26 | 7.34E-25 | ELF4 | 0.43649279 | 12.8083148 | 7.02E-34 | 2.44E-32 |
| DMBT1 | 0.45806119 | 11.2070437 | 5.15E-26 | 1.17E-24 | TNFRSF1A | 0.43643678 | 12.8062846 | 7.17E-34 | 2.48E-32 |
| SPINK5 | 0.45719231 | 11.180161 | 6.55E-26 | 1.46E-24 | MMP2 | 0.43610283 | 12.7941834 | 8.13E-34 | 2.80E-32 |
| RAB31 | -0.4568683 | -11.170148 | 7.16E-26 | 1.59E-24 | C8orf4 | 0.435819 | 12.783903 | 9.05E-34 | 3.10E-32 |
| MASP2 | 0.45680172 | 11.1680894 | 7.29E-26 | 1.61E-24 | PRKD2 | 0.434639 | 12.741215 | 1.41E-33 | 4.75E-32 |
| PIK3C3 | -0.456017 | -11.143861 | 9.05E-26 | 1.96E-24 | MYD88 | 0.43441487 | 12.733116 | 1.54E-33 | 5.15E-32 |
| OPRK1 | 0.45562812 | 11.1318676 | 1.01E-25 | 2.14E-24 | SEC61A1 | 0.43436111 | 12.7311737 | 1.57E-33 | 5.23E-32 |
| SKAP1 | 0.45370379 | 11.0726335 | 1.70E-25 | 3.52E-24 | ALCAM | -0.4341436 | -12.723318 | 1.70E-33 | 5.65E-32 |
| NONO | -0.4535428 | -11.067687 | 1.78E-25 | 3.65E-24 | ACTB | 0.43396743 | 12.716956 | 1.82E-33 | 6.02E-32 |
| CACNA1F | 0.45313998 | 11.0553154 | 1.99E-25 | 4.03E-24 | S100A11 | 0.43139973 | 12.624444 | 4.74E-33 | 1.51E-31 |
| NFATC3 | -0.4528948 | -11.04779 | 2.12E-25 | 4.29E-24 | GPRC5B | -0.4306624 | -12.597949 | 6.23E-33 | 1.97E-31 |
| TCF12 | -0.452274 | -11.028749 | 2.51E-25 | 5.05E-24 | TFRC | 0.42842353 | 12.5176854 | 1.42E-32 | 4.36E-31 |
| ITGAD | 0.45209177 | 11.023163 | 2.64E-25 | 5.27E-24 | C1RL | 0.42815255 | 12.5079901 | 1.57E-32 | 4.78E-31 |
| IL3 | 0.45132534 | 10.9996901 | 3.25E-25 | 6.44E-24 | XBP1 | 0.42776301 | 12.4940601 | 1.82E-32 | 5.49E-31 |
| IL31RA | 0.45114158 | 10.9940668 | 3.41E-25 | 6.72E-24 | TNFRSF18 | 0.4277059 | 12.4920184 | 1.85E-32 | 5.59E-31 |
| LCN2 | 0.44966408 | 10.9489165 | 5.08E-25 | 9.80E-24 | C16orf62 | -0.4273206 | -12.47825 | 2.14E-32 | 6.39E-31 |
| KIR2DL1 | 0.4483938 | 10.9101888 | 7.15E-25 | 1.35E-23 | RAB18 | -0.4271438 | -12.471933 | 2.28E-32 | 6.78E-31 |
| IL1RL1 | 0.448294 | 10.9071499 | 7.34E-25 | 1.38E-23 | RIPK1 | 0.42713114 | 12.4714823 | 2.29E-32 | 6.80E-31 |
| SLC44A2 | -0.4481736 | -10.903485 | 7.58E-25 | 1.43E-23 | TAB1 | -0.4264428 | -12.446912 | 2.95E-32 | 8.66E-31 |
| SLAMF6 | 0.44798311 | 10.8976859 | 7.98E-25 | 1.49E-23 | CKAP4 | 0.42494716 | 12.3936168 | 5.08E-32 | 1.48E-30 |
| IL23A | 0.44708908 | 10.8704974 | 1.01E-24 | 1.87E-23 | IFIT5 | -0.4246562 | -12.383264 | 5.65E-32 | 1.64E-30 |
| PRKD1 | -0.4470802 | -10.870229 | 1.01E-24 | 1.87E-23 | PRDM1 | 0.42254239 | 12.3081847 | 1.21E-31 | 3.44E-30 |
| KIR3DS1 | 0.44705545 | 10.8694753 | 1.02E-24 | 1.88E-23 | CTSB | 0.42212938 | 12.2935441 | 1.41E-31 | 3.97E-30 |
| SH2D1B | 0.44583763 | 10.8325079 | 1.41E-24 | 2.53E-23 | TRIM8 | -0.4214908 | -12.270927 | 1.77E-31 | 4.98E-30 |
| TNFRSF13C | 0.44578072 | 10.8307821 | 1.43E-24 | 2.56E-23 | PSMD12 | 0.42131701 | 12.2647738 | 1.89E-31 | 5.29E-30 |
| CPN2 | 0.44533002 | 10.8171213 | 1.62E-24 | 2.86E-23 | GLB1 | 0.42121493 | 12.2611612 | 1.96E-31 | 5.47E-30 |
| GFI1 | 0.44497029 | 10.8062251 | 1.78E-24 | 3.13E-23 | TGFB3 | 0.42120352 | 12.2607576 | 1.97E-31 | 5.49E-30 |
| BTNL8 | 0.44360327 | 10.7648776 | 2.55E-24 | 4.36E-23 | IL4R | 0.42106562 | 12.2558783 | 2.07E-31 | 5.73E-30 |
| IRAK3 | 0.44328464 | 10.7552537 | 2.77E-24 | 4.69E-23 | SURF4 | 0.42013192 | 12.2228682 | 2.89E-31 | 7.90E-30 |
| PGLYRP3 | 0.44190863 | 10.7137504 | 3.98E-24 | 6.58E-23 | KCNN4 | 0.42006065 | 12.2203503 | 2.96E-31 | 8.09E-30 |
| CPN1 | 0.44143121 | 10.6993726 | 4.51E-24 | 7.39E-23 | TNFRSF10D | 0.41946967 | 12.1994833 | 3.66E-31 | 9.92E-30 |
| TNFAIP1 | -0.4412614 | -10.69426 | 4.71E-24 | 7.68E-23 | LOXL3 | 0.41769065 | 12.1367813 | 6.90E-31 | 1.82E-29 |
| CDH17 | 0.44023676 | 10.6634501 | 6.16E-24 | 9.81E-23 | BCL10 | 0.41723065 | 12.1205961 | 8.12E-31 | 2.12E-29 |
| SERPINB12 | 0.4388707 | 10.6224525 | 8.78E-24 | 1.37E-22 | LTBR | 0.41514389 | 12.0473137 | 1.70E-30 | 4.30E-29 |
| CLNK | 0.43724082 | 10.5736565 | 1.34E-23 | 2.04E-22 | NCSTN | 0.41463153 | 12.0293562 | 2.03E-30 | 5.12E-29 |
| CFHR4 | 0.43716492 | 10.5713875 | 1.37E-23 | 2.08E-22 | MYO1G | 0.4141651 | 12.0130202 | 2.39E-30 | 6.02E-29 |
| IFNA21 | 0.43702369 | 10.5671658 | 1.42E-23 | 2.15E-22 | C1R | 0.41371404 | 11.9972334 | 2.80E-30 | 6.99E-29 |
| CPB2 | 0.43618195 | 10.5420249 | 1.76E-23 | 2.63E-22 | HMOX1 | 0.41302334 | 11.9730804 | 3.57E-30 | 8.82E-29 |
| ABL1 | -0.4354092 | -10.518973 | 2.14E-23 | 3.16E-22 | ARPC2 | 0.4129335 | 11.9699406 | 3.68E-30 | 9.08E-29 |
| MLH1 | -0.4353677 | -10.517737 | 2.17E-23 | 3.19E-22 | MUL1 | 0.41205967 | 11.9394232 | 5.00E-30 | 1.22E-28 |
| PCBP2 | -0.4348571 | -10.502523 | 2.47E-23 | 3.59E-22 | TNFAIP3 | 0.41187183 | 11.9328683 | 5.33E-30 | 1.30E-28 |
| SLC30A8 | 0.43454128 | 10.4931198 | 2.68E-23 | 3.87E-22 | CAPZA1 | 0.41088231 | 11.8983683 | 7.52E-30 | 1.81E-28 |
| BTN3A3 | -0.4344827 | -10.491376 | 2.72E-23 | 3.93E-22 | HSP90B1 | 0.41033157 | 11.8791885 | 9.11E-30 | 2.18E-28 |
| SHMT2 | -0.4343433 | -10.487227 | 2.82E-23 | 4.06E-22 | DUSP3 | 0.40892773 | 11.8303693 | 1.48E-29 | 3.48E-28 |
| PSMD11 | -0.4340048 | -10.477156 | 3.07E-23 | 4.38E-22 | KYNU | 0.40751596 | 11.7813761 | 2.40E-29 | 5.52E-28 |
| TCN1 | 0.43315759 | 10.4519748 | 3.81E-23 | 5.36E-22 | PSMC4 | 0.40672427 | 11.7539463 | 3.15E-29 | 7.17E-28 |
| IGLV4-3 | 0.43231658 | 10.4270113 | 4.72E-23 | 6.55E-22 | PECAM1 | 0.40659996 | 11.7496421 | 3.29E-29 | 7.46E-28 |
| NOTCH1 | -0.4319285 | -10.415503 | 5.21E-23 | 7.17E-22 | LGALS1 | 0.40590816 | 11.7257032 | 4.17E-29 | 9.37E-28 |
| TREML2 | 0.43080488 | 10.3822239 | 6.92E-23 | 9.36E-22 | C5AR1 | 0.40438565 | 11.6731042 | 6.99E-29 | 1.54E-27 |
| EIF2AK2 | -0.4301323 | -10.362332 | 8.20E-23 | 1.10E-21 | GLA | 0.40318905 | 11.6318457 | 1.05E-28 | 2.27E-27 |
| PAG1 | -0.429643 | -10.347873 | 9.27E-23 | 1.23E-21 | FES | 0.40110215 | 11.5600603 | 2.12E-28 | 4.48E-27 |
| ROCK1 | -0.4295267 | -10.344438 | 9.55E-23 | 1.26E-21 | RAP2B | 0.40050252 | 11.539474 | 2.59E-28 | 5.45E-27 |
| CLEC4M | 0.42924564 | 10.3361406 | 1.02E-22 | 1.35E-21 |  | | | | |
| PLA2G3 | 0.42894691 | 10.3273245 | 1.10E-22 | 1.45E-21 |  | | | | |
| CALCOCO2 | -0.4287309 | -10.320952 | 1.17E-22 | 1.53E-21 |  | | | | |
| HRG | 0.42833698 | 10.3093375 | 1.29E-22 | 1.67E-21 |  | | | | |
| SP2 | -0.4281326 | -10.303314 | 1.35E-22 | 1.75E-21 |  | | | | |
| CD300LB | 0.4278112 | 10.2938457 | 1.47E-22 | 1.90E-21 |  | | | | |
| KLK5 | 0.42643163 | 10.2532585 | 2.07E-22 | 2.63E-21 |  | | | | |
| CD200R1 | 0.42610595 | 10.2436895 | 2.25E-22 | 2.84E-21 |  | | | | |
| PUM2 | -0.4257266 | -10.23255 | 2.47E-22 | 3.10E-21 |  | | | | |
| CEACAM1 | 0.42435207 | 10.1922425 | 3.47E-22 | 4.28E-21 |  | | | | |
| PRSS3 | 0.42421919 | 10.1883502 | 3.58E-22 | 4.41E-21 |  | | | | |
| RBM14 | -0.4228677 | -10.14881 | 5.00E-22 | 6.06E-21 |  | | | | |
| RAET1E | 0.42239164 | 10.1349019 | 5.62E-22 | 6.77E-21 |  | | | | |
| POLR3E | -0.4223066 | -10.132418 | 5.74E-22 | 6.90E-21 |  | | | | |
| ESR1 | 0.42150231 | 10.1089471 | 6.99E-22 | 8.33E-21 |  | | | | |
| COLEC10 | 0.42114661 | 10.0985761 | 7.63E-22 | 9.05E-21 |  | | | | |
| KIR2DL4 | 0.42088554 | 10.0909679 | 8.13E-22 | 9.61E-21 |  | | | | |
| TEC | 0.42078942 | 10.0881674 | 8.32E-22 | 9.83E-21 |  | | | | |
| FASLG | 0.42067807 | 10.0849238 | 8.55E-22 | 1.01E-20 |  | | | | |
| NLRP2 | 0.41905922 | 10.0378288 | 1.27E-21 | 1.47E-20 |  | | | | |
| PSMF1 | -0.4190216 | -10.036736 | 1.28E-21 | 1.48E-20 |  | | | | |
| MAGT1 | -0.4189188 | -10.033751 | 1.31E-21 | 1.51E-20 |  | | | | |
| VTCN1 | 0.41865183 | 10.0259954 | 1.40E-21 | 1.61E-20 |  | | | | |
| CFHR5 | 0.41830542 | 10.0159391 | 1.52E-21 | 1.74E-20 |  | | | | |
| SIRPB1 | 0.41790209 | 10.0042371 | 1.68E-21 | 1.91E-20 |  | | | | |
| DCD | 0.41742846 | 9.99050436 | 1.88E-21 | 2.13E-20 |  | | | | |
| CD40LG | 0.41716131 | 9.98276308 | 2.01E-21 | 2.26E-20 |  | | | | |
| PRCP | -0.4166786 | -9.9687831 | 2.25E-21 | 2.51E-20 |  | | | | |
| CAT | -0.4157258 | -9.9412196 | 2.84E-21 | 3.12E-20 |  | | | | |
| RAB10 | -0.4156219 | -9.9382143 | 2.91E-21 | 3.19E-20 |  | | | | |
| GBP6 | 0.41544509 | 9.93310515 | 3.03E-21 | 3.32E-20 |  | | | | |
| PRG3 | 0.41532132 | 9.92952897 | 3.12E-21 | 3.41E-20 |  | | | | |
| BMX | 0.41512925 | 9.92398042 | 3.27E-21 | 3.56E-20 |  | | | | |
| ELF2 | -0.4147595 | -9.9133033 | 3.57E-21 | 3.88E-20 |  | | | | |
| DEFA6 | 0.41465844 | 9.9103868 | 3.66E-21 | 3.96E-20 |  | | | | |
| PAK2 | -0.4146101 | -9.9089906 | 3.70E-21 | 4.00E-20 |  | | | | |
| CREBBP | -0.4144962 | -9.9057047 | 3.81E-21 | 4.10E-20 |  | | | | |
| KIR3DL1 | 0.41446609 | 9.90483571 | 3.83E-21 | 4.13E-20 |  | | | | |
| NPY5R | 0.41339274 | 9.87388953 | 4.95E-21 | 5.24E-20 |  | | | | |
| IDH1 | -0.4130867 | -9.8650749 | 5.33E-21 | 5.60E-20 |  | | | | |
| DIAPH1 | 0.41284643 | 9.85815789 | 5.64E-21 | 5.90E-20 |  | | | | |
| CHST4 | 0.41214631 | 9.83801585 | 6.66E-21 | 6.87E-20 |  | | | | |
| HRH2 | 0.41205383 | 9.83535666 | 6.81E-21 | 7.01E-20 |  | | | | |
| CCL1 | 0.41148308 | 9.8189542 | 7.80E-21 | 7.97E-20 |  | | | | |
| CBFB | -0.4108634 | -9.8011617 | 9.03E-21 | 9.15E-20 |  | | | | |
| HSPD1 | -0.4102858 | -9.7845921 | 1.03E-20 | 1.04E-19 |  | | | | |
| CMTM3 | -0.4099309 | -9.7744167 | 1.12E-20 | 1.12E-19 |  | | | | |
| TRIM25 | -0.4097041 | -9.7679175 | 1.19E-20 | 1.18E-19 |  | | | | |
| CD8A | 0.40948812 | 9.76173127 | 1.25E-20 | 1.24E-19 |  | | | | |
| PRKDC | -0.4088088 | -9.7422846 | 1.46E-20 | 1.44E-19 |  | | | | |
| GPLD1 | 0.40768648 | 9.71019814 | 1.90E-20 | 1.84E-19 |  | | | | |
| SPG21 | -0.407202 | -9.6963645 | 2.13E-20 | 2.05E-19 |  | | | | |
| TRIM10 | 0.40640226 | 9.6735479 | 2.57E-20 | 2.45E-19 |  | | | | |
| RAB3A | 0.40566564 | 9.6525567 | 3.05E-20 | 2.87E-19 |  | | | | |
| BMPR1A | -0.4055098 | -9.648118 | 3.16E-20 | 2.97E-19 |  | | | | |
| PHB | -0.4043744 | -9.6158159 | 4.11E-20 | 3.81E-19 |  | | | | |
| KIR2DL2 | 0.40285669 | 9.57271681 | 5.83E-20 | 5.31E-19 |  | | | | |
| NBN | -0.4019181 | -9.5461108 | 7.23E-20 | 6.49E-19 |  | | | | |
| CLEC4G | 0.40071878 | 9.51216668 | 9.52E-20 | 8.44E-19 |  | | | | |
| BPI | 0.40060523 | 9.50895597 | 9.77E-20 | 8.65E-19 |  | | | | |
| MED1 | -0.4005932 | -9.5086154 | 9.79E-20 | 8.67E-19 |  | | | | |
| OPRM1 | 0.40018277 | 9.49701496 | 1.08E-19 | 9.47E-19 |  | | | | |
|  |  |  |  |  |  |  |  |  |  |
